# Gyrotactic microorganism hybrid nanofluid over a Riga plate subject to activation energy and heat source: numerical approach

**DOI:** 10.1038/s41598-023-27562-y

**Published:** 2023-08-22

**Authors:** Ebrahem A. Algehyne, Anwar Saeed, Muhammad Arif, Muhammad Bilal, Poom Kumam, Ahmed M. Galal

**Affiliations:** 1 Department of Mathematics, Faculty of Science, University of Tabuk, P.O.Box 741, Tabuk, 71491 Saudi Arabia; 2 Nanotechnology Research Unit (NRU), University of Tabuk, Tabuk, 71491 Saudi Arabia; 3 Center of Excellence in Theoretical and Computational Science (TaCS-CoE), Faculty of Science, King Mongkut’s University of Technology Thonburi (KMUTT), 126 Pracha Uthit Rd., Bang Mod, Thung Khru, Bangkok, 10140 Thailand; 4 Department of Mathematics, University of Peshawar, Sheikh Taimur Academic Block-II, Peshawar, 25120 Khyber Pakhtunkhwa Pakistan; 5 Department of Mechanical Engineering, College of Engineering in Wadi Alddawasir, Prince Sattam Bin Abdulaziz University, Wadi Alddawasir, Saudi Arabia; 6 Production Engineering and Mechanical Design Department, Faculty of Engineering, Mansoura University, P.O 35516, Mansoura, Egypt

**Keywords:** Engineering, Mathematics and computing

## Abstract

The current article aims to examine the magnetohydrodynamics (MHD) impact on the flow of MgO–Ag/water-based hybrid nanoliquid with motile microorganisms and the fluid is allowed to flow over a Riga plate subject to slip effects and activation energy. Furthermore, the presence of a uniform heat source/sink is also addressed in the energy equation. In addition to this, the thermophoresis effect is highlighted in the concentration equation. From the present proposed model, we get a non-linear system of the governing equations. The obtained system of partial differential equations (PDEs) is converted to the dimensionless system of ordinary differential equations (ODEs) using the similarity transformation. The obtained high non-linear system of equations has been solved numerically, using the parametric continuation method (PCM). In the present analysis, the main motivation is to highlight the heat transfer rate of MgO–Ag/water-based hybrid nanofluid flow over a Riga plate. The second motivation of the present research is to highlight the impact of slip conditions on the velocity, energy, and mass profiles. From the graphical analysis, it is depicted that the slip conditions reduce the velocity, energy, and mass outlines. From the present analysis, we concluded that volume friction reduced the flow profile while increasing the temperature of the fluid flow over a Riga plate. All the parameters of the present research are highlighted in velocity temperature and concertation of the fluid. In addition to this in all the figures we have compared the hybrid nanofluid with mono nanofluid and the also the comparison between slip and no-slip conditions have carried out through graphs for velocity, temperature, and concentration.

## Introduction

The new advanced mechanism of the magnetic field which can be designed by the adjustment of a cluster of changeless magnets and an alternative electrode over a plane surface is recognized as a “Riga plate”. This advanced arrangement and formulation of the Riga plate in different fluid flow models play a vital role and induced the influence of the Lorentz effect. This advanced mechanism of the Riga plate the first time was formulated by Gailitis and Lielausis^1^ where the authors carried out this research experimentally in the Riga Laboratory. This Riga plate arrangement is very effective and beneficial in different fluid flow problems. The arrangement helps to reduce skin friction in many physical situations specifically in the submarines. The Riga plate has numerous uses in fluid dynamics and different physical phenomena. The key factor magnetic term cast-off in Riga plate then the motion of the fluid in such situation is recognized as Hartmann number. The researchers and scientists investigated the Riga plate and discussed its enormous applications of the Riga plate. Recently, Prabakaran et al.^2^ scrutinized the upshot of CNTs-based nanoliquid flow across a Riga sheet. Grinberg^3^ investigated and carried out research, using the applications of the Riga plate. Similarly, some novel physical insights of the fluid flow across a moving horizontal Riga plate with advanced physical applications are documented by Wakif et al.^4^. In addition to this Rasool et al.^5^ developed a second-grade nanofluid model and considered the flow over a vertically heated Riga plate for some advanced applications. Faizan et al.^6^ addressed the nanoparticles and bioconvective motile microorganisms-based Sutterby nanofluid flow with the entropy analysis over a Riga plate. Rasool and Wakif^7^ where the authors developed the numerical study of the spectral examination of EMHD. In this study, the authors considered second-grade fluid with nanoparticles, and the fluid is considered over a vertical Riga plate. Kayikci et al.^8^, Loganathan et al.^9^ and Prabakaran et al.^2^ investigated the unique properties of using the Riga plate in different physical situations.

Nanofluid is very important in modern sciences and has enormous applications in heat distribution processing phenomena. The systems in which nanofluid is used as a working fluid can have better heat distribution and thermal performance compared to regular fluids. In comparison, the heat communication rate of solids is higher than any kind of fluid. Therefore, the researchers initially, tried to incorporate some solid particles to boost the energy transference rate. Therefore, keeping this motivation in mind Maxwell^10^ experimented by adding microparticles to regular fluids. During the experiment of Maxwell, he found that micro-sized particles failed to enhance the heat transference rate. This experiment failed because the suspension of microparticles makes the fluid unstable and the fluid passing through narrow channels causes the blockage and produces sedimentation in the fluid. After this unsuccessful experiment, Maxwell in 1992 Choi^11^ carried out an experiment in Argon national laboratory he tried to suspend nanosized particles in conventional fluids. During this analysis, he noticed that the addition of nano particulates does not affect the stability of the resultant fluid and also can’t produce sedimentation in the fluid. After this successful experiment, the idea of nanofluid is used in every field of sciences and nanotechnology and has enormous applications in engineering and biological sciences. The research on nanofluid is getting more attention and can be applied nanofluid applications in many real phenomena. Recently, Alrabaiah et al.^12^, inspected the nanofluid its properties, and applications that how nanofluid boost up the heat transfer properties using different nano additives in base fluids. Molana et al.^13^ where the researchers investigated and highlight the influence of thermal properties on nanofluid thermal conduction. Alagumalai et al.^14^ established the conceptual examination of barriers to the nanofluid mechanism and their advance uses in modern science and commercialization. Arif et al.^15^ documented the effect of nano particulates in engine oil for advanced applications in automobiles. Ben et al.^16^ highlighted the unique applications of nanofluids as a cutting fluid in different operating systems and working machinery. Ali et al.^17^ developed a Casson fluid model and considered MoS2 nanoparticles with the consequence of radiation. Iqbal et al.^18^, calculated the buoyancy upshot of the Maxwell nanoliquid flow across a vertical surface. Some other uses of nanoliquid in different fields of science can be found in the references^19–21^.

The suspension of two different nanocomposites in a single base fluid is termed a hybrid nanofluid. As the nanofluid performance was appreciated in many practical situations and produced good thermal conductivity in the working fluids. But still, in modern science and thermal sciences, we need an advanced cooling application with good thermal performance. Therefore, researchers dissolve pairs of nanomaterials in a single base fluid and found that hybrid nanoliquid performance is higher than regular fluids. Due to the increase of hybrid nanofluid, researchers take two and more nanoparticles for high thermal efficiency in different systems. Like, Gholinia et al.^22^ highlighted the investigation of using different base fluids by the uniform dispersion of CNTs hybrid nanoparticles, and the authors consider circular cylinders for the fluid flow. Similarly, Arif et al.^23^, calculated the thermal efficiency of engine oil-based hybrid nanofluid by the uniform dispersion of GO and MoS2 nanoparticles, and the fluid flow is taken along an extending cylinder. In addition to this, the authors calculated the heat transfer rate and concluded that the hybrid nano-liquids are higher than the thermal distribution of nanofluid and regular fluids. Yahya et al.^24^ discussed the impact of MoS2 and ZnO nanoparticles using engine oil base hybrid nanoliquid flow. Lv et al.^25^ discoursed the magnetized hybrid nanoliquid flow in a vertical stretch cylinder and investigated some advanced features of hybrid nanofluid. Rajesh et al.^26^ the authors studied hybrid nanoliquid and calculated the impact on the MHD flow with heat allocation by considering the non-uniform temperature. The suspension of different nanoparticles with various shapes in water-based fluid for advanced cooling applications was reported by Arif et al.^27^. Ali et al.^28^ developed the hybrid nanoliquid flow model and discussed its applications for energy transmission.

Motivated by the unique characteristics and useful applications of heat sources in modern science scholars studied the influence of heat sources on the fluid flow. The heat source is moving fluids and has enormous daily life applications like nuclear reactors, heat turbines, heat generators, and metal waste, in the analysis of reactor safety and combustion processes. Motivated by these applications, many investigators inspected the upshot of heat source-sink in their studies. Chamkha^29^ reported the 2D flow over an elongating sheet with the heat source. Turkyilmazoglu^30^ discussed the MHD impact of natural convection with the mutual result of heat generation/absorption and porous media. Cortell^31^ discussed the heat source effects in the free convection flow. Grosan and Pop^32^ and Postelnicu and Pop^33^ inspected the heat source upshot on the fluid flow across a vertical flat with the characteristics of energy transference.

In boundary layer flow problems, the presence of activation energy plays a key role. The activation energy has useful applications in various physical phenomena like the field of engineering sciences, in the field of oil reservoirs, geothermal, engineering and sciences reservoirs. Khan et al.^34^ and highlighted the axisymmetric rotating Oldroyd-B fluid flow and explained some mechanical applications. Liu and Li^35^ where the authors inspected the activation energy and its useful modern uses. Similarly, Ramesh^36^ studied the nanoliquid flow, by considering the upshot of activation energy. Alqarni et al.^37^, Alsallami et al.^38^, and Khan et al.^39^ where the authors examined the consequences of activation energy in different circumstances.

The motile gyrotactic microorganism has many daily life applications in different fields of science. The gyrotactic microorganism phenomenon is studied by many researchers. Lu et al.^40^ considered the numerical treatment of nanoliquid flow with the combined effect of gyrotactic microbes. Azam et al.^41^ numerically reported the bioconvection and energy implications in the advancement of thermal transmission of chemically reactive Casson fluid over a porous cylinder with the effects of gyrotactic microorganisms and variable viscosity. Azam^42^ demonstrated a theoretical formulation and simulation analysis of the steady bioconvective flow of chemically bonded Sutterby nanoliquid under the upshot of nonlinear radiation and gyrotactic microbes. It was worth noting that the micro-organisms field has been upgraded to allow for a more accurate estimation of the microbe’s difference variable and Peclet number. Furthermore, Azam^43^ reported the bioconvection and activation energy through radiative Williamson nanoliquid flow with gyrotactic microbes and heat flux. Additionally, they inspected the swimming of microbes in the unsteady flow with advanced biomedical applications^25,44–46^.

Motivated from the above-mentioned literature to the best of the author knowledge no work is carried out to highlight the impact of gyrotactic microorganism’s hybrid nanoliquid over a Riga plate subject to activation energy and heat source. The obtained model is solved numerically by applying the PCM methodology. The impact of all the flow constraints is highlighted on the flow heat and mass profiles. In the coming segments, the model is expressed, solved and debated.

## Mathematical formulation

**Figure 1:**
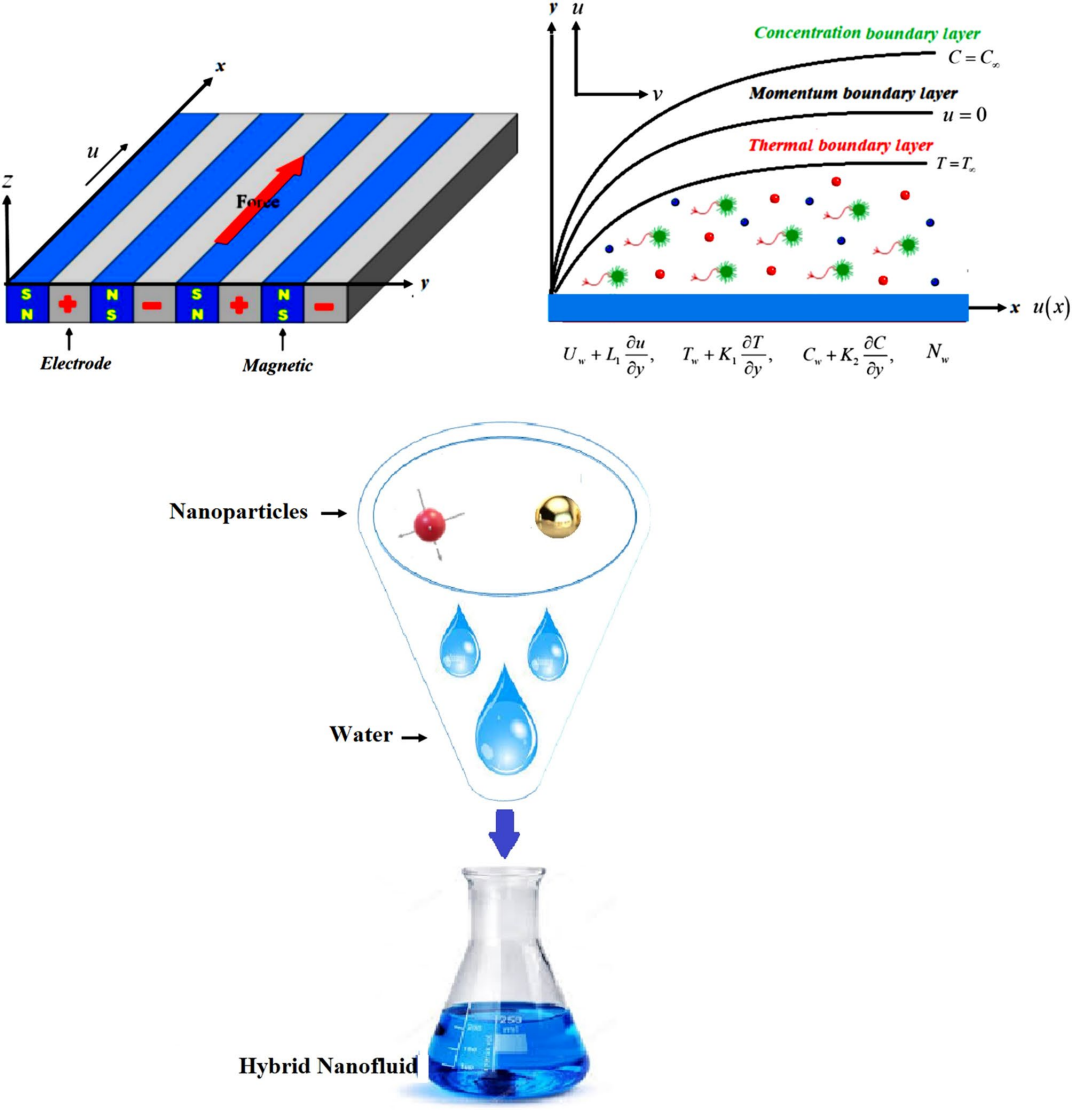
The physical representation of the fluid flow.

In the present research, we have considered the MHD steady 2D nanoliquid (MgO–Ag/water) flow thermal transport characteristics. It has been considered that the Riga plate is disturbed which starts a motion with a free stream velocity $U_{W} = cx$ and the fluid has temperature difference in ambient temperature $T_{\infty }$ and surface temperature $T_{W}$, in a similar way the ambient concentration can be expressed $C_{\infty }$ and the wall concentration can be expressed as $C_{W}$ and finally the uniform and ambient concertation of the gyrotactic microorganism can be expressed as $N_{W}$ and $N_{\infty }$ respectively. The physical draft of the present proposed model is publicized in Fig. 1. The system of equations for the flow heat, concertation of fluid, and gyrotactic microorganism is given as^6,47^:

1 $$\frac{\partial u}{{\partial x}} + \frac{\partial v}{{\partial y}} = 0$$

2 $$u\frac{\partial u}{{\partial x}} + v\frac{\partial v}{{\partial y}} = \frac{{\mu_{hnf} }}{{\rho_{hnf} }}\frac{{\partial^{2} u}}{{\partial y^{2} }} + \frac{{\pi j_{0} M_{0} }}{{8\rho_{hnf} }}\exp \left( { - \frac{\pi }{a}y} \right)$$

3 $$u\frac{\partial T}{{\partial x}} + v\frac{\partial T}{{\partial y}} = \frac{{k_{hnf} }}{{(\rho Cp)_{hnf} }}\frac{{\partial^{2} T}}{{\partial y^{2} }} - \frac{{Q_{0} }}{{(\rho Cp)_{hnf} }}(T - T_{\infty } )$$

4 $$u\frac{\partial C}{{\partial x}} + v\frac{\partial C}{{\partial y}} = D_{hnf} \frac{{\partial^{2} C}}{{\partial y^{2} }} - \frac{\partial }{\partial y}\left( {V_{T} \left( {C - C_{\infty } } \right)} \right) - K_{r}^{2} \left( {\frac{T}{{T_{\infty } }}} \right)^{n} \exp \left( {\frac{{ - E_{a} }}{\kappa T}} \right)\left( {C - C_{\infty } } \right),$$

5 $$u\frac{\partial N}{{\partial x}} + v\frac{\partial N}{{\partial y}} + \frac{{bW_{C} }}{{\left( {C - C_{\infty } } \right)}}\left[ {\frac{\partial }{\partial y}\left( {N\frac{\partial C}{{\partial y}}} \right)} \right] = D_{m} \frac{{\partial^{2} N}}{{\partial y^{2} }}$$

Thermophoretic velocity is given as^47^:

$$V_{T} = - \frac{Kvf}{{T_{r} }}(T - T_{\infty } ).$$

Here it can be noticed that $K$ is thermophoretic coefficient and the physical boundary conditions to be satisfied are given as under:

6 $$\begin{gathered} u = U_{w} + L_{1} \frac{\partial u}{{\partial y}},\,\,v = 0,\,\,T = T_{w} + K_{1} \frac{\partial T}{{\partial y}}, \hfill \\ C = C_{w} + K_{2} \frac{\partial C}{{\partial y}},\,\,\,N = N_{w} \hfill \\ \end{gathered}$$

7 $$u = 0,\,\,\,C \to C_{\infty } ,\,\,\,T \to T_{\infty } ,\,\,N \to N_{\infty } .\;{\text{as}}\;y \to \infty$$

where $U_{w} = cx,\,\,T_{w} = T_{\infty } + b\left( \frac{x}{l} \right)^{2} ,\,\,C_{w} = C_{\infty } + d\left( \frac{x}{l} \right)^{2} ,\,\,l$ denote the plate length, $L_{1}$ denotes velocity,$K_{1}$ thermal slip, and $K_{2}$ is the expression of concentration slip factors.

Similarity variables for the given proposed model are given as under:

8 $$\begin{gathered} \psi = x\sqrt {cv_{f} } f\left( \eta \right),\,\,\eta = \sqrt {\frac{c}{{v_{f} }}} y,\,\,\,u = cxf^{/} \left( \eta \right),\,\,v = - \sqrt {cv_{f} } f\left( \eta \right),\,\,\theta \left( \eta \right) = \frac{{T - T_{\infty } }}{{T_{w} - T_{\infty } }}, \hfill \\ \varphi \left( \eta \right) = \frac{{C - C_{\infty } }}{{C_{W} - C_{\infty } }},\,\,\hbar \left( \eta \right) = \frac{{N - N_{\infty } }}{{N_{W} - N_{\infty } }}, \hfill \\ \end{gathered}$$

By employing Eq. (8) in (1)–(5), we get:

9 $$\frac{{f^{\prime\prime\prime}\left( \eta \right)}}{{A_{1} A_{2} }} + f^{\prime\prime}\left( \eta \right)f\left( \eta \right) - f^{\prime}\left( \eta \right)^{2} + \frac{Q}{{A_{2} }}\exp \left( { - \eta \beta } \right) = 0$$

10 $$\frac{{k_{hnf} }}{{k_{f} }}\frac{{\theta^{\prime\prime}\left( \eta \right)}}{Pr} + A_{3} f\left( \eta \right)\theta^{\prime}\left( \eta \right) - S\theta \left( \eta \right) = 0$$

11 $$A_{1} \varphi^{\prime\prime}\left( \eta \right) + Scf\left( \eta \right)\varphi^{\prime}\left( \eta \right) - \tau Sc\left( {\theta^{\prime\prime}\left( \eta \right)\varphi \left( \eta \right) + \theta^{\prime}\left( \eta \right)\varphi^{\prime}\left( \eta \right)} \right) - \omega \left( {\theta \Delta + 1} \right)^{n} \varphi e^{{\frac{ - A}{{\left( {1 + \Delta \theta } \right)}}}} = 0,$$

12 $$\hbar^{\prime\prime} + L_{b} f\hbar^{\prime} - Pe\left( {\varphi^{\prime}\hbar^{\prime} + \varphi^{\prime\prime}\left( {\sigma_{1} + \hbar } \right)} \right) = 0$$

Boundary conditions (6–7) are reduced into:

13 $$\begin{gathered} f^{\prime}\left( 0 \right) = 1 + S_{1} f^{\prime\prime}\left( 0 \right),\,\,f\left( 0 \right) = 0,\,\,\,\theta \left( 0 \right) = 1 + S_{2} \,\theta^{\prime}\left( 0 \right),\,\,\,\varphi \left( 0 \right) = 1 + S_{3} \varphi^{\prime}\left( 0 \right),\,\,\hbar \left( 0 \right) = 1\,\,\,{\text{at}}\,\,\eta = 0 \hfill \\ f^{\prime}\left( \infty \right) = 0,\,\,\,\theta \left( \infty \right) = 0,\,\,\varphi \left( \infty \right) = 0,\,\,\hbar \left( \infty \right) = 0\,\,\,{\text{as}}\,\,\eta \to \infty . \hfill \\ \end{gathered}$$

In the above Eqs. (9)–(11), $A_{1} ,\,\,A_{2} ,\,\,A_{3}$ are expressed as:

$$A_{1} = \frac{{\mu_{hnf} }}{{\mu_{bf} }},\,\,\,\,A_{2} = \frac{{\rho_{hnf} }}{{\rho_{bf} }},\,\,\,A_{3} = \frac{{(\rho C_{p} )_{hnf} }}{{(\rho C_{p} )_{bf} }}.$$

Here, $Q = \frac{{\pi j_{0} M_{0} }}{{8\rho_{f} U_{w} c}}$ is local Hartmann number, $\beta = \sqrt {\frac{{\pi^{2} vf}}{{\rho f^{C} }}}$ is the dimensionless parameter, $\Delta = \frac{{T_{w} - T_{\infty } }}{{T_{\infty } }}$ is the temperature difference, $Pr = \frac{{\mu_{f} Cpf}}{{k_{f} }}$ is Prandtl number, $Sc = \frac{vf}{{Df}}$ is Schmidt number,$\tau = - \frac{{K\left( {T_{w} - T_{\infty } } \right)}}{{T_{r} }}$ is the thermophoretic parameter, $A = \frac{{ - E_{a} }}{\kappa T}$ is the activation energy, $\omega = \frac{{K_{r}^{2} - L^{2} }}{\upsilon }$ is the chemical reaction, $S = \frac{{Q_{0} }}{{\rho_{f} Cp_{f} c}}$ is the heat source term, $\sigma_{1} = \frac{{N_{\infty } }}{{\left( {N_{W} - N_{\infty } } \right)}}$ the bioconvection constant, $L_{b} = \frac{vf}{{D_{m} }}$ is the Lewis number,$P_{e} = \frac{{bW_{C} }}{{D_{m} }}$ is the Peclet number. $S_{1} = L_{1} \sqrt{\frac{c}{vf}} ,\,S_{2} = K_{1} \sqrt{\frac{c}{vf}}$ and $S_{3} = K_{2} \sqrt{\frac{c}{vf}}$ are the velocity, thermal and concentration slip parameters, respectively.

Some important engineering values can be expressed as follows:

14 $$C_{{_{f} }} = \frac{{\tau_{w} }}{{\rho_{f} U_{w}^{2} }},\,\,\,Nu_{x} = \frac{{xq_{w} }}{{k_{f} \left( {T_{w} - T_{\infty } } \right)}},\,\,\,Sh_{x} = \frac{{xq_{m} }}{{D_{f} \left( {C_{w} - C_{\infty } } \right)}},\,\,Nn_{x} = \frac{{xq_{n} }}{{D_{f} \left( {N_{w} - N_{\infty } } \right)}}.$$

The terms $\tau_{x} ,\,\,q_{m} ,\,\,q_{w}$ and $q_{n}$ are described as:

15 $$\tau_{x} = \mu_{hnf} \left( {\frac{\partial u}{{\partial y}}} \right)_{y = 0} ,\,\,\,q_{w} = - k_{hnf} \left( {\frac{\partial T}{{\partial y}}} \right)_{y = 0} ,\,\,\,q_{m} = - D_{hnf} \left( {\frac{\partial C}{{\partial y}}} \right)_{y = 0} ,\,\,\,q_{n} = - D_{hnf} \left( {\frac{\partial N}{{\partial y}}} \right)_{y = 0} .$$

16 $$\left. \begin{gathered} Re^{0.5} Cf_{x} = \frac{{\mu_{hnf} }}{{k_{f} }}f^{\prime\prime}\left( 0 \right),\,\,Re^{ - 0.5} Nu_{x} = - \frac{{k_{hnf} }}{{k_{f} }}\theta^{\prime}\left( 0 \right),\,\,Re^{ - 0.5} Sh_{x} = - \left( {1 - \phi_{1} } \right)^{2.5} \left( {1 - \phi_{2} } \right)^{2.5} \varphi^{\prime}\left( 0 \right), \hfill \\ Re^{ - 0.5} Nn_{x} = - \left( {1 - \phi_{1} } \right)^{2.5} \left( {1 - \phi_{2} } \right)^{2.5} \hbar^{\prime}\left( 0 \right) \hfill \\ \end{gathered} \right\}$$

## Numerical solution

The fundamental steps using parametric continuation methods are as follows^48,49^:

- *Step 1* Simplification to 1st order ODE
  17 $$\begin{gathered} \mathchar'26\mkern-10mu\lambda _{1} (\eta ) = f(\eta ),\,\,\,\mathchar'26\mkern-10mu\lambda _{2} (\eta ) = f^{\prime}(\eta ),\,\,\,\mathchar'26\mkern-10mu\lambda _{3} (\eta ) = f^{\prime\prime}(\eta ),\,\,\,\mathchar'26\mkern-10mu\lambda _{4} (\eta ) = \theta (\eta ),\,\,\mathchar'26\mkern-10mu\lambda _{5} (\eta ) = \theta ^{\prime}(\eta ), \hfill \\ \mathchar'26\mkern-10mu\lambda _{6} (\eta ) = \varphi (\eta ),\,\,\mathchar'26\mkern-10mu\lambda _{7} (\eta ) = \varphi ^{\prime}(\eta ),\,\,\mathchar'26\mkern-10mu\lambda _{8} (\eta ) = \hbar (\eta ),\,\,\mathchar'26\mkern-10mu\lambda _{9} (\eta ) = \hbar ^{\prime}(\eta ). \hfill \\ \end{gathered}$$
  By putting Eq. (17) in Eq. (9)–(12) and (13), we get:
  18 $$\frac{{\mathchar'26\mkern-10mu\lambda ^{\prime}_{3} \left( \eta \right)}}{{A_{1} A_{2} }} + \mathchar'26\mkern-10mu\lambda _{3} \left( \eta \right)\mathchar'26\mkern-10mu\lambda _{1} \left( \eta \right) - \mathchar'26\mkern-10mu\lambda _{2} \left( \eta \right)^{2} + \frac{Q}{{A_{2} }}\exp \left( { - \eta \beta } \right) = 0$$
  19 $$\frac{{\mathchar'26\mkern-10mu\lambda ^{\prime}_{3} \left( \eta \right)}}{{A_{1} A_{2} }} + \mathchar'26\mkern-10mu\lambda _{3} \left( \eta \right)\mathchar'26\mkern-10mu\lambda _{1} \left( \eta \right) - \mathchar'26\mkern-10mu\lambda _{2} \left( \eta \right)^{2} + \frac{Q}{{A_{2} }}\exp \left( { - \eta \beta } \right) = 0$$
  20 $$A_{1} \mathchar'26\mkern-10mu\lambda ^{\prime}_{7} \left( \eta \right) + Sc\mathchar'26\mkern-10mu\lambda _{1} \left( \eta \right)\mathchar'26\mkern-10mu\lambda _{7} \left( \eta \right) - \tau Sc\left( {\mathchar'26\mkern-10mu\lambda ^{\prime}_{5} \left( \eta \right)\mathchar'26\mkern-10mu\lambda _{6} \left( \eta \right) + \mathchar'26\mkern-10mu\lambda _{5} \left( \eta \right)\mathchar'26\mkern-10mu\lambda _{7} \left( \eta \right)} \right) - \omega \left( {\mathchar'26\mkern-10mu\lambda _{4} \Delta + 1} \right)^{n} \mathchar'26\mkern-10mu\lambda _{6} e^{{\frac{{ - A}}{{\left( {1 + \Delta \mathchar'26\mkern-10mu\lambda _{4} } \right)}}}} = 0,$$
  21 $$\mathchar'26\mkern-10mu\lambda ^{\prime}_{9} + L_{b} \mathchar'26\mkern-10mu\lambda _{1} \mathchar'26\mkern-10mu\lambda _{9} - Pe\left( {\mathchar'26\mkern-10mu\lambda _{7} \mathchar'26\mkern-10mu\lambda _{9} + \mathchar'26\mkern-10mu\lambda ^{\prime}_{7} \left( {\sigma _{1} + \hbar } \right)} \right) = 0$$
  The boundary conditions are:
  22 $$\left. \begin{gathered} \mathchar'26\mkern-10mu\lambda _{2} \left( 0 \right) = 1 + S_{1} \mathchar'26\mkern-10mu\lambda _{3} \left( 0 \right),\,\,\,\mathchar'26\mkern-10mu\lambda _{1} \left( 0 \right) = 0,\,\,\,\mathchar'26\mkern-10mu\lambda _{4} \left( 0 \right) = 1 + S_{2} \,\mathchar'26\mkern-10mu\lambda _{5} \left( 0 \right),\,\,\,\mathchar'26\mkern-10mu\lambda _{6} \left( 0 \right) = 1 + S_{3} \mathchar'26\mkern-10mu\lambda _{7} \left( 0 \right),\,\,\mathchar'26\mkern-10mu\lambda _{8} \left( 0 \right) = 1\,\,at\,\,\eta = 0 \hfill \\ \mathchar'26\mkern-10mu\lambda _{2} \left( \infty \right) = 0,\,\,\,\mathchar'26\mkern-10mu\lambda _{4} \left( \infty \right) = 0,\,\,\mathchar'26\mkern-10mu\lambda _{6} \left( \infty \right) = 0,\,\,\mathchar'26\mkern-10mu\lambda _{8} \left( \infty \right) = 0\,\,\,as\,\,\eta \to \infty . \hfill \\ \end{gathered} \right\}$$
- *Step 2* Presenting parameter *p* in Eqs. (18)–(21):
  23 $$\frac{{\mathchar'26\mkern-10mu\lambda ^{\prime}_{3} \left( \eta \right)}}{{A_{1} A_{2} }} + \left( {\mathchar'26\mkern-10mu\lambda _{3} \left( \eta \right) - 1} \right)p\,\mathchar'26\mkern-10mu\lambda _{1} \left( \eta \right) - \mathchar'26\mkern-10mu\lambda _{2} \left( \eta \right)^{2} + \frac{Q}{{A_{2} }}\exp \left( { - \eta \beta } \right) = 0$$
  24 $$\frac{{k_{{hnf}} }}{{k_{f} }}\frac{{\mathchar'26\mkern-10mu\lambda ^{\prime}_{5} \left( \eta \right)}}{{\Pr }} + A_{3} \mathchar'26\mkern-10mu\lambda _{1} \left( \eta \right)\left( {\mathchar'26\mkern-10mu\lambda _{5} \left( \eta \right) - 1} \right)p - S\mathchar'26\mkern-10mu\lambda _{4} \left( \eta \right) = 0$$
  25 $$A_{1} \mathchar'26\mkern-10mu\lambda ^{\prime}_{7} \left( \eta \right) + Sc\mathchar'26\mkern-10mu\lambda _{1} \left( \eta \right)\left( {\mathchar'26\mkern-10mu\lambda _{7} \left( \eta \right) - 1} \right)p - \tau Sc\left( {\mathchar'26\mkern-10mu\lambda ^{\prime}_{5} \left( \eta \right)\mathchar'26\mkern-10mu\lambda _{6} \left( \eta \right) + \mathchar'26\mkern-10mu\lambda _{5} \left( \eta \right)\mathchar'26\mkern-10mu\lambda _{7} \left( \eta \right)} \right) - \omega \left( {\mathchar'26\mkern-10mu\lambda _{4} \Delta + 1} \right)^{n} \mathchar'26\mkern-10mu\lambda _{6} e^{{\frac{{ - A}}{{\left( {1 + \Delta \mathchar'26\mkern-10mu\lambda _{4} } \right)}}}} = 0,$$
  26 $$\mathchar'26\mkern-10mu\lambda ^{\prime}_{9} \left( \eta \right) + L_{b} \mathchar'26\mkern-10mu\lambda _{1} \left( \eta \right)\left( {\mathchar'26\mkern-10mu\lambda _{9} \left( \eta \right) - 1} \right)p - Pe\left( {\mathchar'26\mkern-10mu\lambda _{7} \left( \eta \right)\mathchar'26\mkern-10mu\lambda _{9} \left( \eta \right) + \mathchar'26\mkern-10mu\lambda ^{\prime}_{7} \left( \eta \right)\left( {\sigma _{1} + \hbar } \right)} \right) = 0$$

## Results and discussion

The impact of the motile gyrotactic microbe and Ag-MgO/ water-base hybrid nanoliquid flow over a Riga plate is highlighted in this section. The system of the equation of the proposed model is given in Eqs. (2)–(5) are transformed by applying the similarity variables we get the transform system given in Eqs. (9)–(12). The non-linear equations are handled by finding the numerical solutions using the parametric continuation method. In addition to this, the absence and presence of the slip parameters $S_{1}$ on velocity, $S_{2}$ temperature, and $S_{3}$ concentration fields are highlighted, and compared their results through graphs. All the pertinent parameters like a magnetic dimensionless parameter $\beta$, Hartmann number $Q$, volume friction $\phi$, Schmidt number $Sc$, heat source $S$, activation energy $A$, chemical reaction $\omega$, thermophoretic parameter $\tau$, and Peclet number $Pe$.

The geometry of the proposed model is given in Fig. 1. The impact of constraints on the flow profile is portrayed in Figs. 2, 3 and 4. The impact of parameters of interest on temperature distribution is highlighted in Figs. 5, 6 and 7. The consequences of constraints on the mass profile of the fluid are highlighted in Figs. 8, 9, 10, 11, 12, 13 and 14. Finally, the effect of $Pe$ and volume friction $\phi$ on the motile gyrotactic microbe profile is shown in Fig. 14.

**Figure 2:**
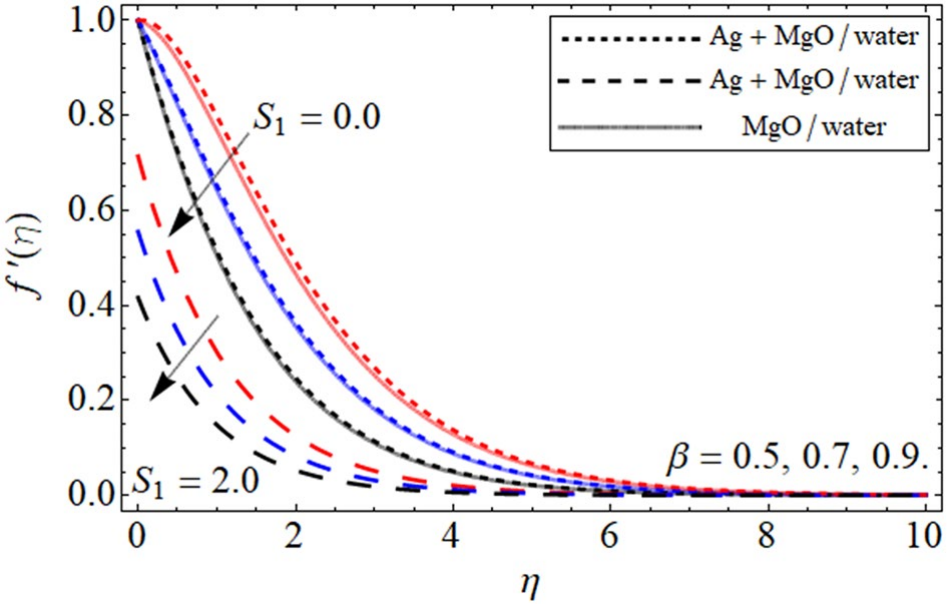
$$f^{\prime}\left( \eta \right)$$ versus $$\beta$$. When $$\phi_{1} = \phi_{2} = 0.01,$$ $$Q = 0.1,$$ $$S = 0.2,$$ $$S_{2} = 0.2,$$ $$S_{1} = 0.1,$$ $$S_{3} = 0.1,$$ $$A = 0.3,$$ $$\omega = 0.3,$$ $$Sc = 0.7,$$ $$\tau = 0.4,$$ $$Pe = 0.3,$$ $$Lb = 0.1.$$

**Figure 3:**
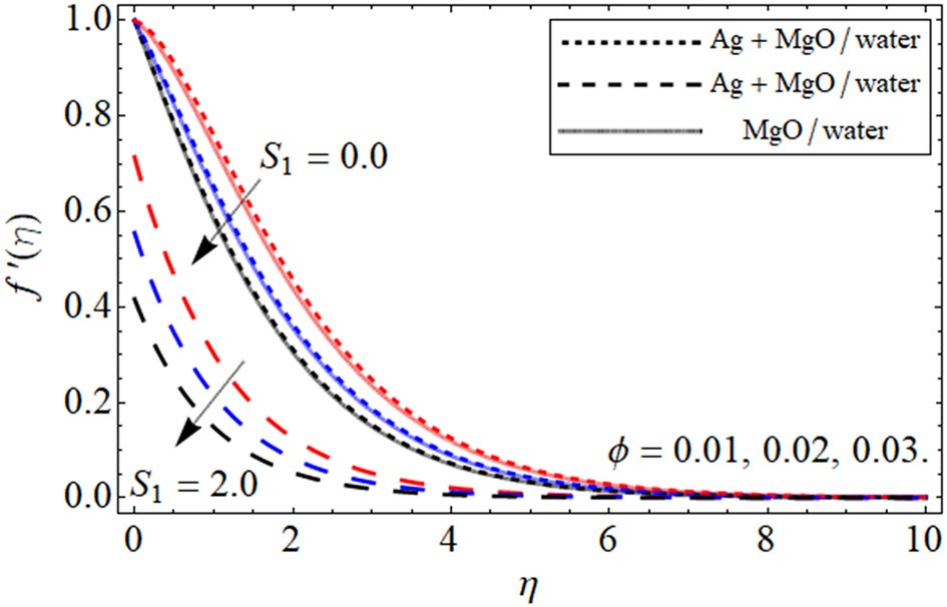
$$f^{\prime}\left( \eta \right)$$ versus $$\phi$$. When $$\beta = 0.5,$$ $$Q = 0.1,$$ $$S = 0.2,$$ $$S_{2} = 0.2,$$ $$S_{1} = 0.1,$$ $$S_{3} = 0.1,$$ $$A = 0.3,$$ $$\omega = 0.3,$$ $$Sc = 0.7,$$ $$\tau = 0.4,$$ $$Pe = 0.3,$$ $$Lb = 0.1.$$

**Figure 4:**
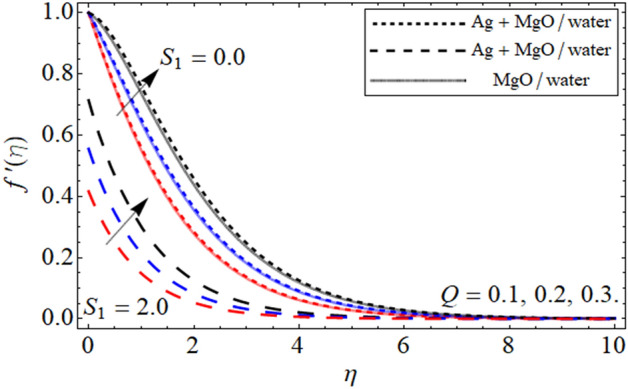
$$f^{\prime}\left( \eta \right)$$ versus $$Q$$. When $$\phi_{1} = \phi_{2} = 0.01,$$ $$\beta = 0.5,$$ $$S = 0.2,$$ $$S_{2} = 0.2,$$ $$S_{1} = 0.1,$$ $$S_{3} = 0.1,$$ $$A = 0.3,$$ $$\omega = 0.3,$$ $$Sc = 0.7,$$ $$\tau = 0.4,$$ $$Pe = 0.3,$$ $$Lb = 0.1.$$

**Figure 5:**
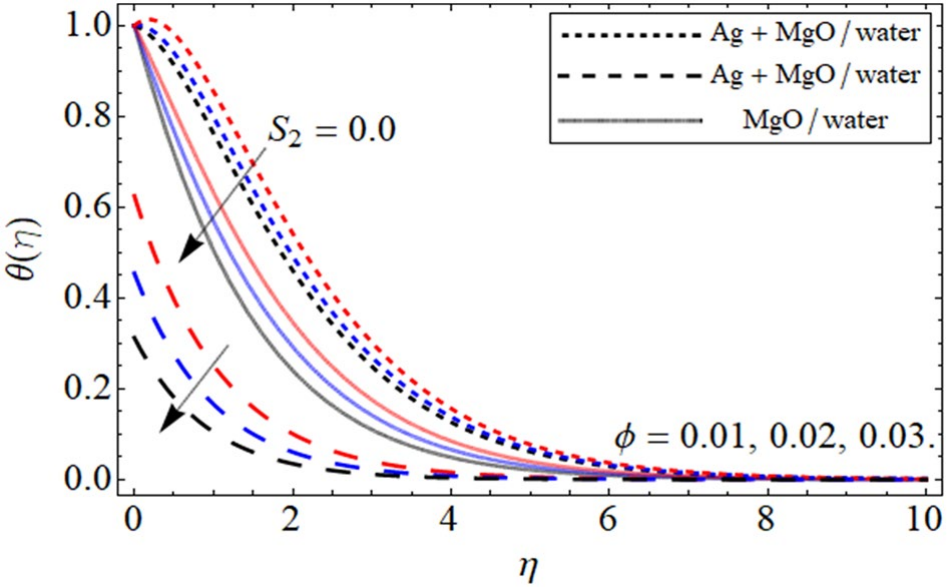
$$\theta \left( \eta \right)$$ versus $$\phi$$. When $$\beta = 0.5,$$ $$Q = 0.1,$$ $$S = 0.2,$$ $$S_{2} = 0.2,$$ $$S_{1} = 0.1,$$ $$S_{3} = 0.1,$$ $$A = 0.3,$$ $$\omega = 0.3,$$ $$Sc = 0.7,$$ $$\tau = 0.4,$$ $$Pe = 0.3,$$ $$Lb = 0.1.$$

**Figure 6:**
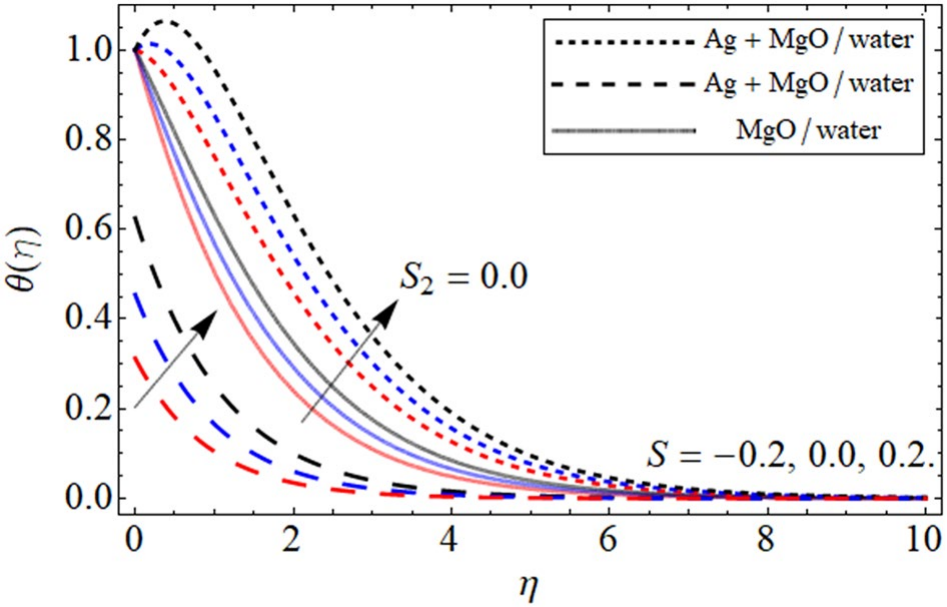
$$\theta \left( \eta \right)$$ versus $$S$$. When $$\phi_{1} = \phi_{2} = 0.01,$$ $$\beta = 0.5,$$ $$Q = 0.1,$$ $$S_{2} = 0.2,$$ $$S_{1} = 0.1,$$ $$S_{3} = 0.1,$$ $$A = 0.3,$$ $$\omega = 0.3,$$ $$Sc = 0.7,$$ $$\tau = 0.4,$$ $$Pe = 0.3,$$ $$Lb = 0.1.$$

**Figure 7:**
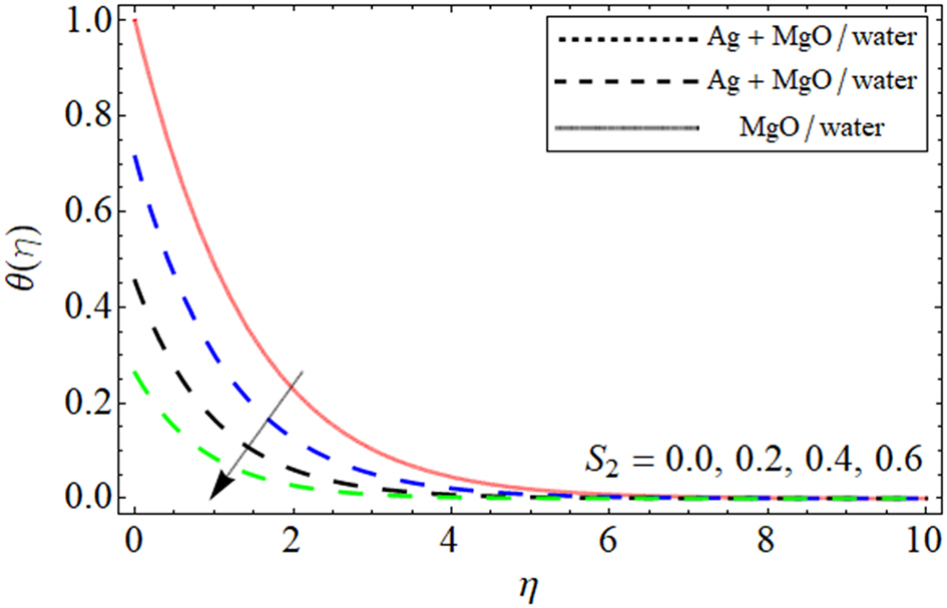
$$\theta \left( \eta \right)$$ versus $$S_{2}$$. When $$\phi_{1} = \phi_{2} = 0.01,$$ $$\beta = 0.5,$$ $$Q = 0.1,$$ $$S = 0.2,$$ $$S_{3} = 0.1,$$ $$S_{1} = 0.1,$$ $$A = 0.3,$$ $$\omega = 0.3,$$ $$Sc = 0.7,$$ $$\tau = 0.4,$$ $$Pe = 0.3,$$ $$Lb = 0.1.$$

**Figure 8:**
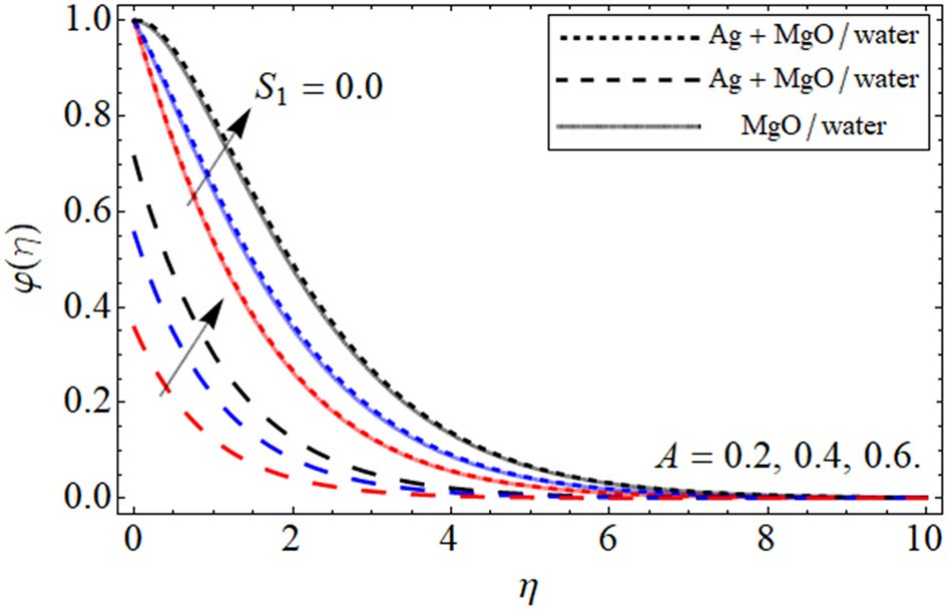
$$\varphi \left( \eta \right)$$ versus $$A$$. When $$\phi_{1} = \phi_{2} = 0.01,$$ $$\beta = 0.5,$$ $$Q = 0.1,$$ $$S = 0.2,$$ $$S_{2} = 0.2,$$ $$S_{3} = 0.1,$$ $$S_{1} = 0.1,$$ $$\omega = 0.3,$$ $$Sc = 0.7,$$ $$\tau = 0.4,$$ $$Pe = 0.3,$$ $$Lb = 0.1.$$

**Figure 9:**
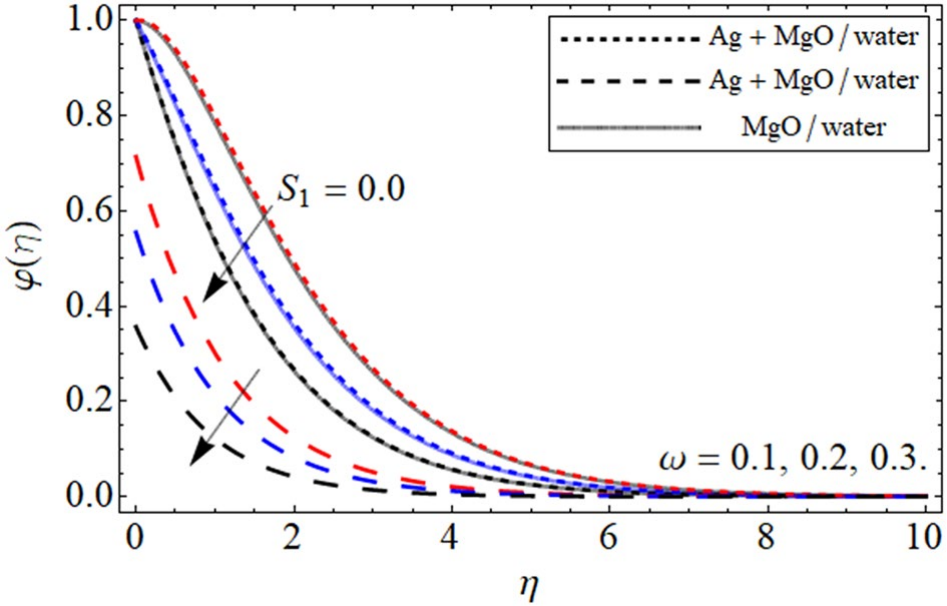
$$\varphi \left( \eta \right)$$ versus $$\omega$$. When $$\phi_{1} = \phi_{2} = 0.01,$$ $$\beta = 0.5,$$ $$Q = 0.1,$$ $$S = 0.2,$$ $$S_{2} = 0.2,$$ $$S_{1} = 0.1,$$ $$S_{3} = 0.1,$$ $$A = 0.3,$$ $$Sc = 0.7,$$ $$\tau = 0.4,$$ $$Pe = 0.3,$$ $$Lb = 0.1.$$

**Figure 10:**
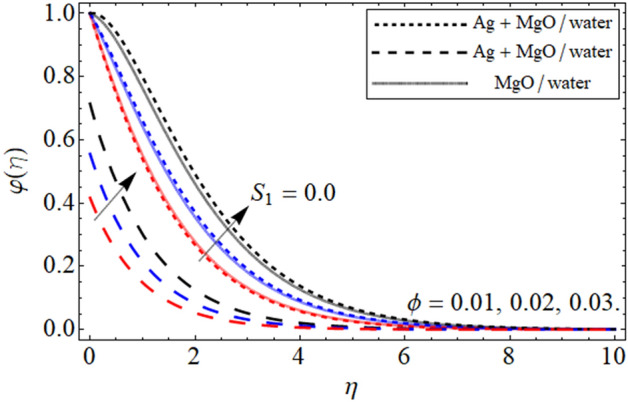
$$\varphi \left( \eta \right)$$ versus $$\phi$$. When $$\beta = 0.5,$$ $$Q = 0.1,$$ $$S = 0.2,$$ $$S_{2} = 0.2,$$ $$S_{1} = 0.1,$$ $$S_{3} = 0.1,$$ $$A = 0.3,$$ $$\omega = 0.3,$$ $$Sc = 0.7,$$ $$\tau = 0.4,$$ $$Pe = 0.3,$$ $$Lb = 0.1.$$

**Figure 11:**
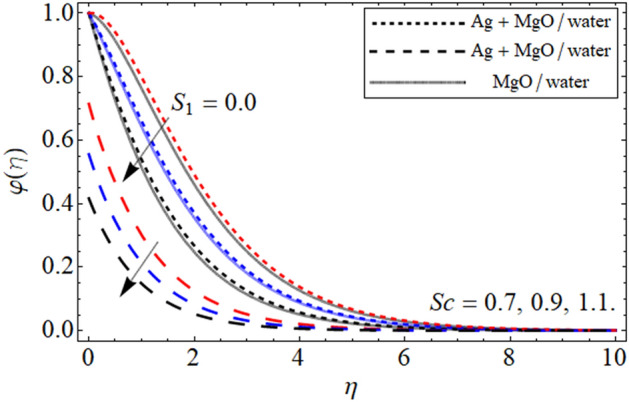
$$\varphi \left( \eta \right)$$ versus $$Sc$$. When $$\phi_{1} = \phi_{2} = 0.01,$$ $$\beta = 0.5,$$ $$Q = 0.1,$$ $$S = 0.2,$$ $$S_{2} = 0.2,$$ $$S_{1} = 0.1,$$ $$S_{3} = 0.1,$$ $$A = 0.3,$$ $$\omega = 0.3,$$ $$\tau = 0.4,$$ $$Pe = 0.3,$$ $$Lb = 0.1.$$

**Figure 12:**
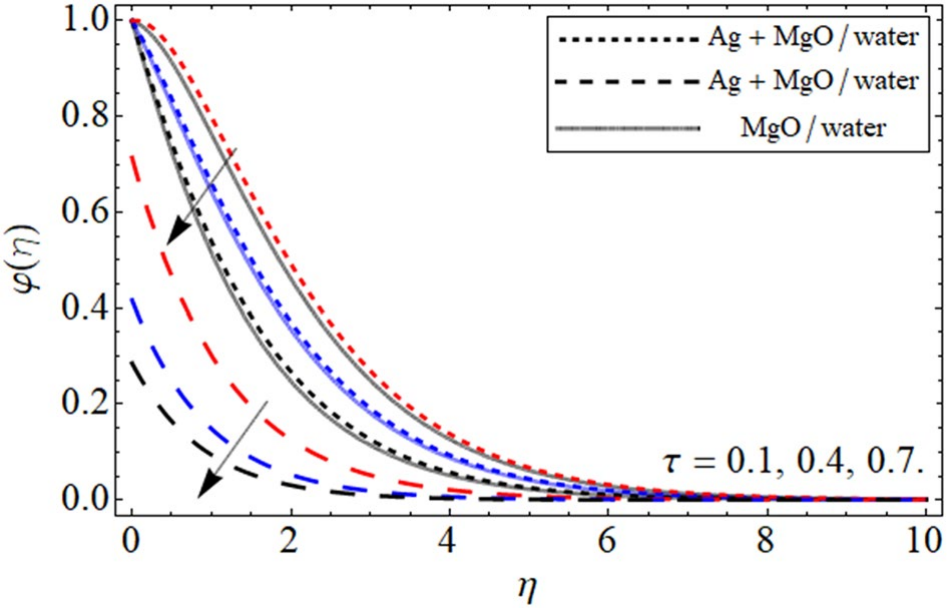
$$\varphi \left( \eta \right)$$ versus $$\tau$$. When $$\phi_{1} = \phi_{2} = 0.01,$$ $$\beta = 0.5,$$ $$Q = 0.1,$$ $$S = 0.2,$$ $$S_{2} = 0.2,$$ $$S_{1} = 0.1,$$ $$S_{3} = 0.1,$$ $$A = 0.3,$$ $$\omega = 0.3,$$ $$Sc = 0.7,$$ $$Pe = 0.3,$$ $$Lb = 0.1.$$

**Figure 13:**
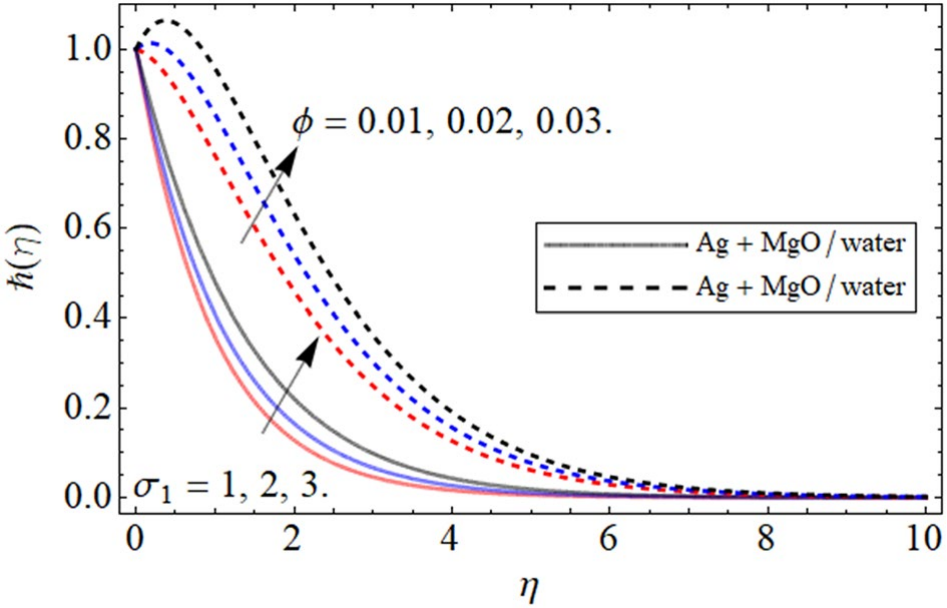
$$\hbar \left( \eta \right)$$ versus $$\phi$$. When $$\phi_{1} = \phi_{2} = 0.01,$$ $$\beta = 0.5,$$ $$Q = 0.1,$$ $$S = 0.2,$$ $$S_{2} = 0.2,$$ $$S_{1} = 0.1,$$ $$S_{3} = 0.1,$$ $$A = 0.3,$$ $$\omega = 0.3,$$ $$Sc = 0.7,$$ $$\tau = 0.4,$$ $$Pe = 0.3,$$ $$Lb = 0.1.$$

**Figure 14:**
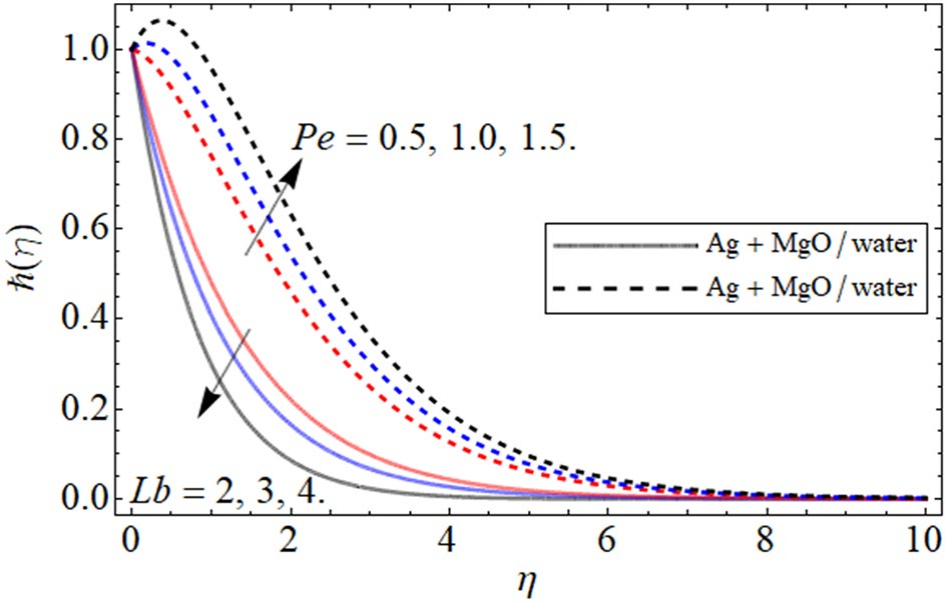
$$\hbar \left( \eta \right)$$ versus $$Pe\,\,{\text{and}}\,\,Lb$$. When $$\phi_{1} = \phi_{2} = 0.01,$$ $$\beta = 0.5,$$ $$Q = 0.1,$$ $$S = 0.2,$$ $$S_{2} = 0.2,$$ $$S_{1} = 0.1,$$ $$S_{3} = 0.1,$$ $$A = 0.3,$$ $$\omega = 0.3,$$$$Sc = 0.7,$$ $$\tau = 0.4.$$

### Flow characteristics

Figure 2 elucidates the upshot of $\beta$ the velocity curve $f^{\prime}\left( \eta \right)$. This fig is plotted for the velocity profile in which we carried out a comparison between the slip condition ($S_{1} > 0$)and no-slip condition ($S_{1} = 0$) on the velocity profile. It is worth noting that in all the graphs the comparison between Ag-MgO/water-based hybrid nanoliquid with MgO/water base nanofluid is highlighted. From the present figure, it is depicted that the growing values $\beta$ decrease the fluid velocity in both cases with slip and no-slip conditions of velocity. This decrease occurs in the fluid velocity due the resistive forces developed within the fluid for higher values of $\beta$ due to which the fluid velocity declines. Moreover, the comparative analysis of hybrid nanoliquid and mono nanofluid shows that the thermal presentation of hybrid nanoliquid shows good results than nanofluid and regular fluid.

The impact of $\phi$ against $f^{\prime}\left( \eta \right)$ is inspected in Fig. 3. From the fig a decrease is seen in the magnitude of the velocity in both the cases of slip and no-slip conditions. This decrease in the fluid velocity is due to the fact that higher $\phi$ concentration in the fluid increases due to which the viscous forces developed and retard the fluid motion over the Riga plate. The impact of the Hartman number $Q$ demonstrated in Fig. 4. From the fig it is depicted that the improving values of $Q$ upsurges in the fluid velocity it is because of higher values of $Q$ reduced resistive forces due to which the viscosity within the fluid declines as a result of the velocity of the fluid increases in both the cases slip and no-slip condition.

### Heat transfer characteristics

This section provides the assessment of the energy curve against different embedded parameters. The flow parameters the effect of which are highlighted on the temperature profile are volume friction $\phi$, heat source-sink $S$, and the thermal slip effect $S_{2}$ are highlighted in the figures. The impact of volume friction $\phi$ on temperature distribution is plotted in Fig. 5. From the fig it can be detected that flourishing values of $\phi$ the energy of the fluid gets high it is because when the nanoparticles concentration increases it make the fluid temperature high because the concertation produces resistive and fractional forces which are the main causes for the enhancement in temperature. In addition to this, we plotted the comparison between the slip and no-slip conditions of the temperature profile. This is to notify that in all the figures the comparison is carried out between hybrid nanofluid and nanofluid. The impact of the heat source $S$ is visualized in Fig. 6. During the analysis of the heat source-sink $S$ on the energy outline it is found that higher the values of $S$ the temperature get high it is because the main motivation behind considering the heat source-sink $S$ in our proposed model is to increase the energy curve. Figure 7 depicts the slip effect $S_{2}$ on the temperature. The growing values of $S_{2}$ decreases the thermal distribution profile. This decrease in the temperature of the system is due the slip conditions in the temperature distribution.

### Mass transfer characteristics

The aim of the present section is to explain the significance of different embedding constraints on concentration profiles. Figures 8, 9, 10, 11 and 12 displayed the impact of different embedding factor on the mass transference. The presence of activation energy $A$ is portrayed in Fig. 8. The mass curve enhances with the growing values of $A$. This increment in the mass curve is due the increase in $A$ because is helpful in the increase in mass profile. The influence of chemical reaction factor $\omega$ is emphasized in Fig. 9. Increasing the values of $\omega$ declines the concentration distribution of the fluid. The physical sketch of the chemical reaction parameter clearly shows the decreasing behavior in the fluid concentration. In addition, the effect of concertation slip effect $S_{3}$ is also highlighted. In both the cases the effect of $\omega$ is same. And the comparison of hybrid nanoliquid with the nanofluid are also addressed in different physical situation.

The impact of $\phi$ on mass outline is displayed in Fig. 10. It is clearly observed that cumulative values of $\phi$ increases the concentration profile. This is physically, correct because increasing the magnitude of concentration of nanoparticles will increase the concentration of the fluid. This impact of $\phi$ is highlighted in both the no slip and slip case. Furthermore, the concentration profiles have been compared for hybrid nanofluid and nanofluid and noticed that hybrid nanoliquid thermal performance is more operative. Figure 11 portrayed the presence of $Sc$ on the concentration of the fluid. From the figure one can visualize that for the greater values of $Sc$ the concentration profile gets lower. The impact of thermophoresis parameter $\tau$ is portrayed in Fig. 12. This fig shows that increasing the values of $\tau$ will off-course decline the mass outline. This decrease occurs in the mass outline with the increase in the $\tau$ because it develops and enhance the fluid concentration. The impact of $\tau$ is highlighted in both the slip and no slip conditions.

### Motile gyrotactic microorganism characteristics

In this section we provide the impact of volume fraction parameter and Peclet numebr Pe is revealed in Figs. 13 and 14 respectively. The cocnetration effect on the gyrotactic miroorgansim is highlighted in Fig. 13. We can see that the gyrotact microorganism enlarge with increasing volume fraction. The figure is plotted to compare the gyrotactic miroorganism with slip and no slip conditons and in both the case the influce of volume fraction isi same. The main motivation of the present is to highlight the comparatve analysis between hybris nanofluid and nanofluid. Keeping this motivation in mind in the gyrotactic microorganism profile is exposed in both cases. The impact of Peclect number and and *Lb* is emphasized in Fig. 14. It can be seen that the increasing values of Peclect numebr *Pe* increases the gyrotactic microbe profile while the higher values of *Lb* decreasee the gyrotactic microorganism profile.

Table 1 exhibits the numerical tentative values of MgO**,** Ag and water. The basic model cast-off for the simulation of the hybrid nanoliquid is offered through Table 2. Table 3 provide the relative estimation of the existing results versus the circulated literature for legitimacy purpose. It can be detected that the current outcomes are correct and consistent. Table 4 demonstrates the numerical assessment of $Re^{ - 1/2} \;Nu_{x}$ (Nusselt number), $- Re^{1/2} \;Cf_{x}$ (drag force), $Re^{ - 1/2} \;Nn_{x}$ (density of motile microbes) and $Re^{ - 1/2} \;Sh_{x}$ (Sherwood number) versus physical entities. It can be detected that the energy communication rate drops with the mounting impact of magnetic field.

**Table 1:** The experimental values of Ag, MgO and water^50,51^.

	$\rho \;(kg/m^{3} )$	$C_{p} \;(j/kgK)$	$k\;(W/mK)$	$\beta_{T} \times 10^{5} \;\left( {{\text{k}}^{ - 1} } \right)$	$\sigma \;\left( {{\text{S}}/{\text{m}}} \right)$
Water	997.1	4179	0.613	$21 \times 10^{ - 5}$	0.05
${\text{MgO}}$	3560	955	45	1.26	$1.42 \times 10^{ - 3}$
${\text{Ag}}$	10,500	235	429	1.89	-

**Table 2:** The mathematical model of hybrid nanoliquid $\left( {\,\phi_{1} = \phi_{MgO} ,\,\,\,\phi_{2} = \phi_{Ag} } \right)$^50,51^.

Properties	Models
Viscosity	$\frac{{\mu_{hnf} }}{{\mu_{bf} }} = \frac{1}{{\left( {1 - \phi_{MgO} - \phi_{Ag} } \right)^{2} }}$
Density	$\frac{{\rho_{hnf} }}{{\rho_{bf} }} = \phi_{MgO} \left( {\frac{{\rho_{MgO} }}{{\rho_{bf} }}} \right) + \phi_{Ag} \left( {\frac{{\rho_{Ag} }}{{\rho_{bf} }}} \right) + \left( {1 - \phi_{MgO} - \phi_{Ag} } \right)$
Thermal Capacity	$\frac{{(\rho C_{p} )_{hnf} }}{{(\rho C_{p} )_{bf} }} = \phi_{MgO} \left( {\frac{{(\rho C_{p} )_{MgO} }}{{(\rho C_{p} )_{bf} }}} \right) + \phi_{Ag} \left( {\frac{{(\rho C_{p} )_{Ag} }}{{(\rho C_{p} )_{bf} }}} \right) + \left( {1 - \phi_{MgO} - \phi_{Ag} } \right)$
Thermal Expansion	$\frac{{(\rho \beta_{T} )_{hnf} }}{{(\rho \beta_{T} )_{bf} }} = \phi_{MgO} \left( {\frac{{(\rho \beta_{T} )_{MgO} }}{{(\rho \beta_{T} )_{bf} }}} \right) + \phi_{Ag} \left( {\frac{{(\rho \beta_{T} )_{Ag} }}{{(\rho \beta_{T} )_{bf} }}} \right) + \left( {1 - \phi_{MgO} - \phi_{Ag} } \right)$
Thermal Conductivity	$\frac{{k_{hnf} }}{{k_{bf} }} = \left[ {\frac{{\left( {\frac{{\phi_{MgO} k_{MgO} + \phi_{Ag} k_{Ag} }}{{\phi_{MgO} + \phi_{Ag} }}} \right) + 2k_{bf} + 2\left( {\phi_{MgO} k_{MgO} + \phi_{Ag} k_{Ag} } \right) - 2\left( {\phi_{MgO} + \phi_{Ag} } \right)k_{bf} }}{{\left( {\frac{{\phi_{MgO} k_{MgO} + \phi_{Ag} k_{Ag} }}{{\phi_{MgO} + \phi_{Ag} }}} \right) + 2k_{bf} - 2\left( {k_{MgO} \phi_{MgO} + k_{Ag} \phi_{Ag} } \right) + 2\left( {\phi_{MgO} + \phi_{Ag} } \right)k_{bf} }}} \right]$
Electrical Conductivity	$\frac{{\sigma_{hnf} }}{{\sigma_{bf} }} = \left[ {\frac{{\left( {\frac{{\phi_{MgO} \sigma_{MgO} + \sigma_{Ag} \phi_{Ag} }}{{\phi_{Ag} + \phi_{MgO} }}} \right) + 2\sigma_{bf} + 2\left( {\phi_{MgO} \sigma_{MgO} + \phi_{Ag} \sigma_{Ag} } \right) - 2\left( {\phi_{MgO} + \phi_{Ag} } \right)\sigma_{bf} }}{{\left( {\frac{{\phi_{MgO} \sigma_{MgO} + \phi_{Ag} \sigma_{Ag} }}{{\phi_{MgO} + \phi_{Ag} }}} \right) + 2\sigma_{bf} - \left( {\phi_{MgO} \sigma_{MgO} + \phi_{Ag} \sigma_{Ag} } \right) + \left( {\phi_{MgO} + \phi_{Ag} } \right)\sigma_{bf} }}} \right]$

**Table 3:** The validation of results versus available studies. When $\phi_{2} = 0,$ $Q = 0.0,$ $S_{1} = 0.0.$

Parameter	Akbar et al.^52^	Chu et al.^53^	Madhukesh et al.^54^	Present results
$\phi_{1}$	$- f^{\prime\prime}\left( 0 \right)$	$- f^{\prime\prime}\left( 0 \right)$	$- f^{\prime\prime}\left( 0 \right)$	$- f^{\prime\prime}\left( 0 \right)$
0.00	1.00000	1.00000	1.00000	1.00000
0.01	1.42421	1.42423	1.42421	1.41438
0.02	2.45948	2.45946	2.45948	2.44962

**Table 4:** The numerical estimation of the drag coefficient, Nusselt number, density of motile microbes and Sherwood number. $\phi_{2} = 0.01,$ $S = 0.2,$ $S_{2} = 0.2,$ $S_{1} = 0.1,$ $S_{3} = 0.1,$ $A = 0.3,$ $\omega = 0.3$.

$\phi$	*Q*	$\beta$	*Sc*	$\tau$	$- Re^{1/2} \;Cf_{x}$	$Re^{ - 1/2} \;Nu_{x}$	$Re^{ - 1/2} \;Sh_{x}$	$Re^{ - 1/2} \;Nn_{x}$
0.01					0.02977	0.00174	0.14286	0.20376
0.02					0.03963	0.02497	0.13606	0.27271
0.03					0.05018	0.04988	0.12948	0.17675
	0.2				0.04787	0.00098	0.16048	0.24843
	0.4				0.06319	0.00016	0.15513	0.19614
	0.6	0.2			0.02977	0.05174	0.16139	0.19858
		0.4			0.02977	0.08192	0.16555	0.20328
		0.6			0.02977	0.00174	0.16593	0.20376
			− 0.5		0.02977	0.00174	0.16593	0.20376
			0.0		0.02977	0.00174	0.12409	0.20581
			0.5		0.02977	0.00174	0.10334	0.20726
				0.1	0.02977	0.00174	0.15828	0.30647
				0.3	0.02977	0.00174	0.16337	0.30892
				0.5	0.02977	0.00174	0.17036	0.31222

## Conclusions

This section provides some major outcomes from the given proposed model. In the present research we have reported water as a base fluid and Ag and MgO nanoparticles are added to form a hybrid nano composite for efficient thermal performance. The present proposed model is transforming by applying the similarity variables. In the present study we focused to highlight all the embedded parameters on the fluid motion over Riga plate., temperature of the fluid concentration and gyrotactic microorganism profile.

Finally, we obtained the following main results from the present research.

- The rising values of solid volume fraction of hybrid nanocomposite will improve the temperature and concentration while decrease the fluid velocity.
- The comparison between hybrid nanofluid and nanofluid is carried in the present research in all the flow heat and concentration configuration and found that hybrid nanofluid have shown good thermal results compared to regular and nanofluid.
- In all the physical configuration the slip and no slip conditions are highlighted and compared their obtained results through graphical analysis.
- The greater values of $Q$ increases the fluid velocity while MHD decreases the fluid velocity.
- The temperature gets higher for the growing values of heat source sink $S$.
- The thermos-phoretic parameter decline the concentration profile.
- The increasing effect has been noticed in gyrotactic microorganism profile for the greater values of *Pe*, while decreasing effect is perceived in the gyrotactic microbe profile for the higher values of *Lb*.
- The present mathematical model can be further extended by using different sorts of fluid and physical effects, and can also be resolved through other numerical, fractional and analytical and techniques.

## Data Availability

All data used in this manuscript have been presented within the article.

## List of symbols

$U_{W}$: Free stream velocity
$$C_{W}$$: Wall concentration
$$V_{T}$$: Thermophoretic velocity
$$K_{r}^{2}$$: Chemical reaction
$$K_{1}$$: Thermal slip
$$L_{1}$$: Slip velocity
*Q*: Hartmann number
$$\Delta$$: Temperature difference
Sc: Schmidt number
*A*: Activation energy factor
$$\sigma_{1}$$: Bioconvection constant
$$\mu_{hnf}$$: Viscosity $${\text{Kgm}}^{ - 1} \;{\text{s}}^{ - 1}$$
$$\rho_{hnf}$$: Density $${\text{Kgm}}^{ - 3}$$
MHD: Magneto hydrodynamics
Ag: Silver
MgO: Magnesium oxide
2D: Two-dimension
$$k_{hnf}$$: Thermal conductivity
$$T_{W}$$: Surface temperature [K]
$$K$$: Thermophoretic coefficient
$$E_{a}$$: Activation energy
$$l$$: Plate length
$$K_{2}$$: Concentration slip
$$Q_{0}$$: Heat source
$$\beta$$: Dimensionless parameter
Pr: Prandtl number
$$\tau$$: Thermophoretic parameter
$\omega$: Chemical reaction term
$L_{b}$: Bioconvection Lewis number
$P_{e}$: Bioconvection Peclet number
$S_{1}$: Slip velocity factor
$S_{2}$: Thermal slip parameter
$S_{3}$: Concentration slip parameter
$$(\rho C_{p} )$$: Thermal capacity
$$(\rho \beta_{T} )$$: Thermal expansion
$$\sigma_{hnf}$$: Electrical conductivity
